# Hyperbaric oxygen therapy for primary sternal osteomyelitis: a case report

**DOI:** 10.1186/1752-1947-7-167

**Published:** 2013-06-27

**Authors:** Tales Rubens de Nadai, Rosemary Furlan Daniel, Mariane Nunes de Nadai, José Joaquim Ribeiro da Rocha, Omar Féres

**Affiliations:** 1 Department of Surgery and Anatomy, Ribeirão Preto Faculty of Medicine, University of São Paulo, Brazil Avenida Bandeirantes, 3900, Ribeirão Preto, SP, 14048-900, Brazil

**Keywords:** Hyperbaric Oxygen Therapy, Primary Osteomyelitis, Sternum

## Abstract

**Introduction:**

Primary osteomyelitis of the sternum is a rare condition, which accounts for 0.3% of all cases of osteomyelitis reported in the literature. The diagnosis requires a high degree of suspicion and confirmation by percutaneous biopsy. The treatment consists of resection of the periosteum and affected bone. Despite reports of successful conservative treatment using antibiotics alone, early surgical intervention plus bacterial control is the definitive treatment; it reduces morbidity, and is the most cost-effective approach for the patient. We report a case of primary osteomyelitis surgically treated with debridement and antibiotics, followed by hyperbaric oxygen therapy.

**Case presentation:**

A 39-year-old Brazilian man without a significant medical history presented with primary osteomyelitis. After a normal chest radiograph and normal laboratory test results, he was treated with 2 weeks of nonsteroidal anti-inflammatory drugs. One month later a presumptive diagnosis of Tietze syndrome was made and he was prescribed prednisolone (60mg/day) for 3 weeks. The following month he presented to our service with swelling, redness, and warmth in the area between his left third and fourth ribs. Subsequent magnetic resonance imaging revealed a large collection of liquid (8.8×6.8×20.2cm) in his chest wall, between the body and the manubrium of the sternum. An area of soft, friable tissue with a large amount of pus was found in his sternum during surgical debridement. Subsequent treatment consisted of antibiotic therapy using metronidazole and cefotaxime plus hyperbaric oxygen therapy. On postoperative day 10 the incision was sutured. The patient was discharged on postoperative day 15 on a regimen of oral ciprofloxacin, and completed hyperbaric oxygen therapy as an out-patient.

**Conclusions:**

The satisfying outcome of this patient reflects the quick action to promote surgical debridement and use of antibiotics, which are both recommended treatments. The closure of the wound in 10 days after debridement suggests that the hyperbaric oxygen therapy might have indirectly, but not conclusively, aided in the premature closure of the wound, avoiding a longer healing by second intention or muscle flap rotation closure.

## Introduction

Primary osteomyelitis of the sternum is a rare condition, which accounts for 0.3% of all cases of osteomyelitis reported in the literature [1]. It differs from secondary osteomyelitis, which results from complications of sternotomy. The primary form affects mainly young people and may be associated with intravenous drug use, trauma, or a central access catheter placed in the subclavian vein. The causative organisms are bacteria and, occasionally, fungi [2,3].

The onset is usually insidious, and the condition is slowly progressive, characterized by edema, redness, tenderness, mass development and pain radiating to the shoulder. The differential diagnosis includes cellulitis, abscess, and bone or soft tissue tumors. The diagnosis requires a high degree of suspicion and confirmation by percutaneous biopsy.

The treatment of primary as well as secondary sternum osteomyelitis consists of resection of the periosteum and affected bone. Unless grossly infected, the posterior periosteum must be kept intact to preserve the mediastinal integrity [4]. Despite reports of successful conservative treatment using antibiotics alone, early surgical intervention plus bacterial control is the definitive treatment; it reduces morbidity, and is the most cost-effective approach for the patient [5]. After debridement, options described in the literature for wound closure are secondary intention closure or muscle flap rotation [6,7]. We report a case of primary osteomyelitis surgically treated with debridement and antibiotics, followed by hyperbaric oxygen therapy (HBOT).

## Case presentation

**Figure 1:**
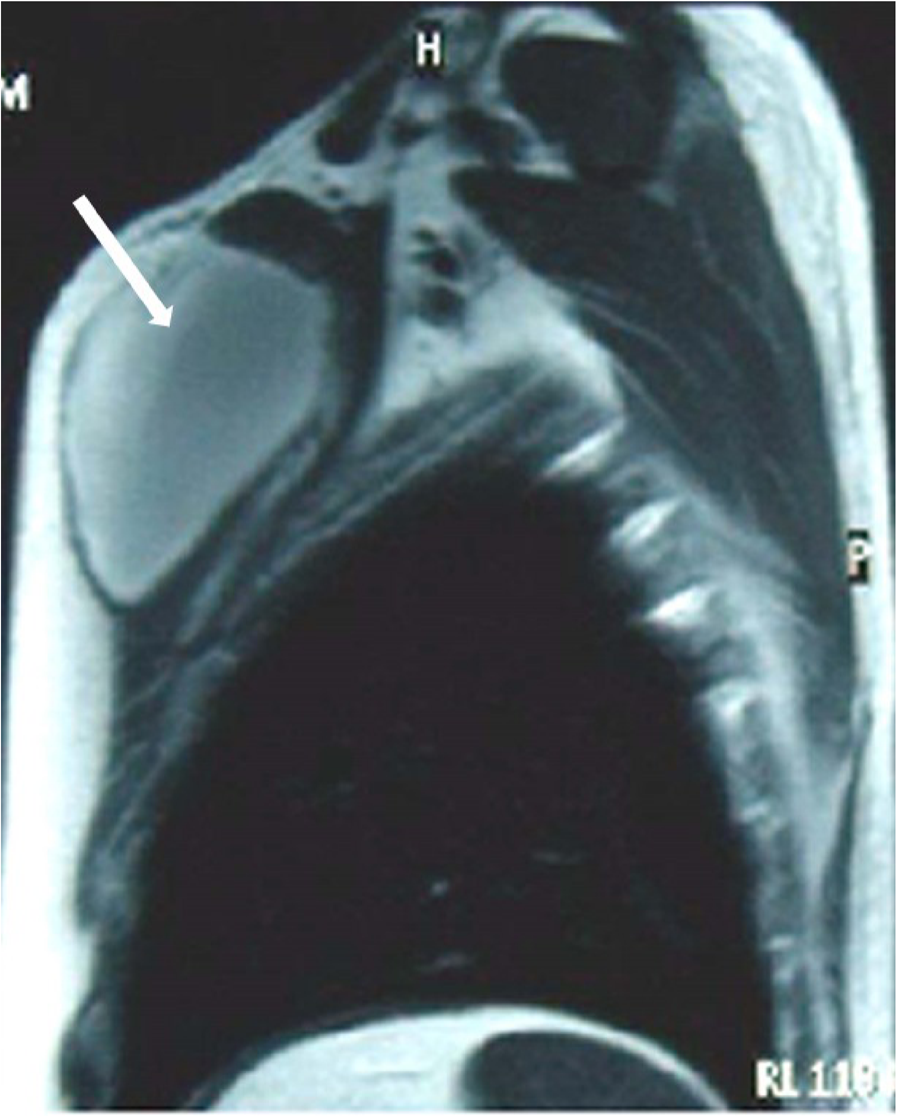
Magnetic resonance image: the arrow shows a large collection of pus in the chest wall located between the body and the manubrium of the sternum.

Two years ago, a 39-year-old Brazilian man without a significant medical history presented with fever and pain at the left costosternal junctions of his third and fourth ribs. After a normal chest radiograph and normal laboratory test results, he was treated with 2 weeks of nonsteroidal anti-inflammatory drugs. One month later there was no improvement, and chest radiography and laboratory testing were again unremarkable. He was then prescribed prednisolone (60mg/day) for 3 weeks, for a presumptive diagnosis of Tietze syndrome. The following month he presented to our service with the same symptoms, along with swelling, redness, and warmth in the area between his left third and fourth ribs. Subsequent magnetic resonance imaging revealed a large collection of fluid (8.8×6.8×20.2cm) in his chest wall, between the body and the manubrium of the sternum (Figure 1).

**Figure 2:**
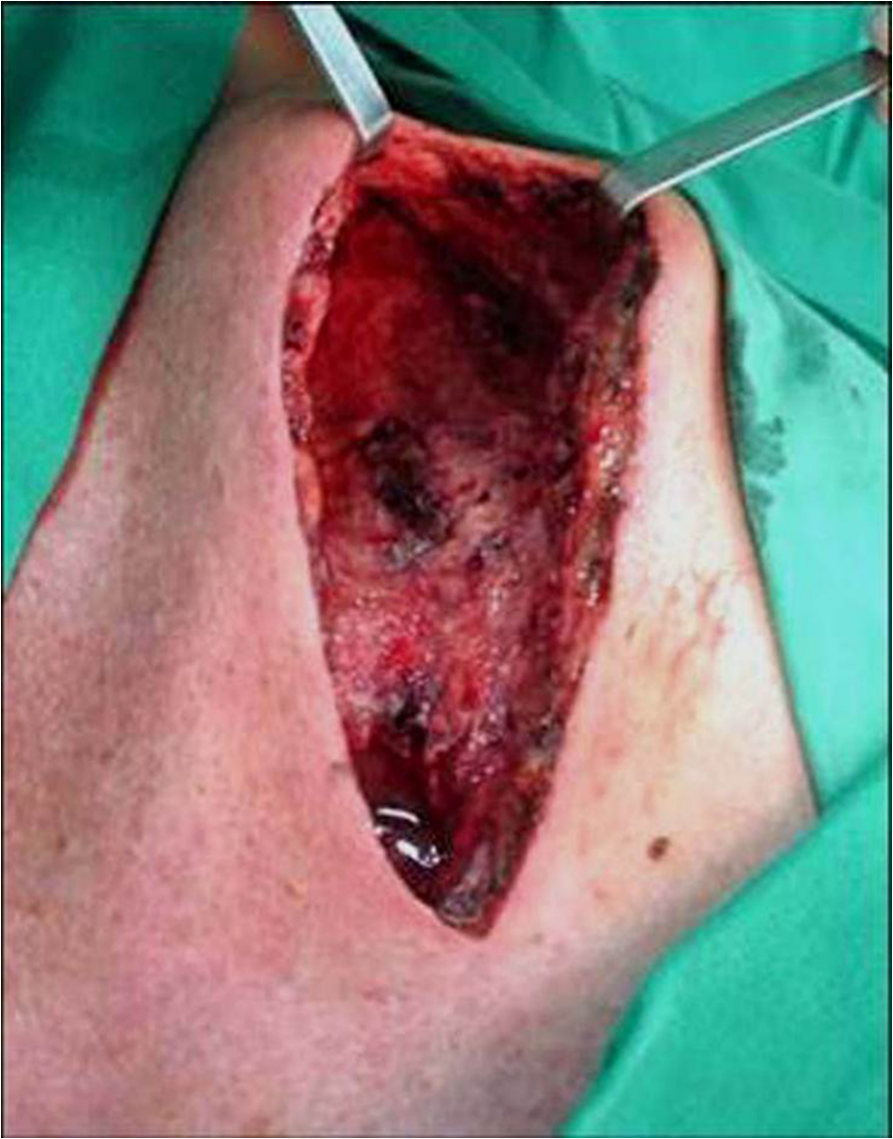
Final appearance after debridement.

**Figure 3:**
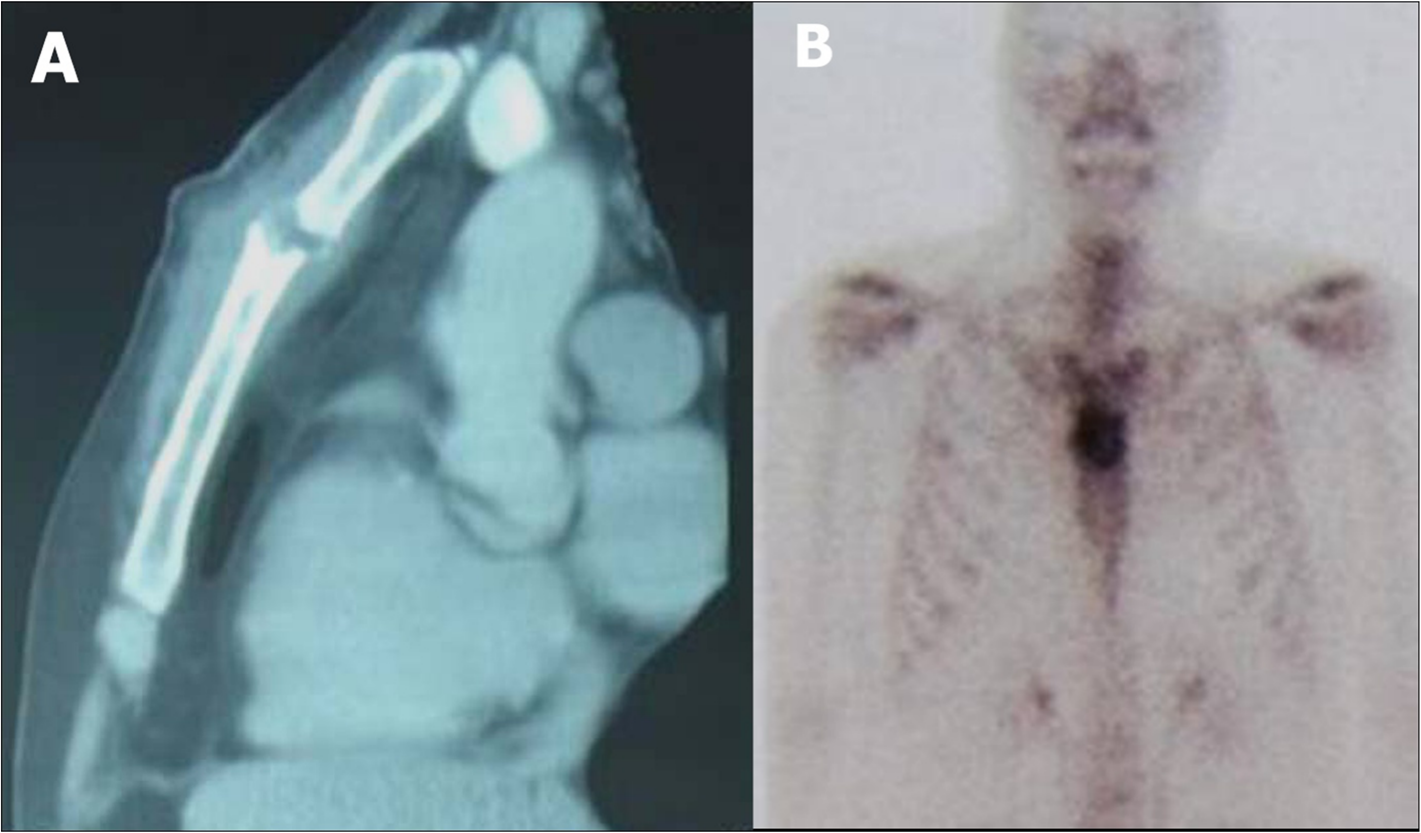
**Day 30 postoperative. A**: Chest computed tomography showing involution of bone injury. **B**: Bone scintigram showing moderate increase in osteoblastic activity in the sternum associated with moderate-to-marked increase in capillary permeability, suggesting bone remodeling and absence of osteomyelitis.

An area of soft, friable tissue with a large amount of pus was found in his sternum during surgical debridement (Figure 2). His posterior sternal wall was conserved, a sample was taken for culture, and the wound was kept open. Subsequent treatment consisted of antibiotic therapy using metronidazole and cefotaxime plus HBOT (monoplace hyperbaric chambers at a pressure between 2 and 3 atmospheric pressure, 1 session per day for 60 days), plus three changes of dressings performed under anesthesia. On postoperative day 10 (tenth session of HBOT), the incision was sutured. Osteomyelitis was confirmed on histopathology, and the culture showed growth of *Staphylococcus aureus*. The patient was discharged on postoperative day 15 on a regimen of oral ciprofloxacin, and completed HBOT as an out-patient. On postoperative day 30, chest computed tomography and bone scintigram (Figure 3) showed bone remodeling and absence of osteomyelitis.

## Discussion

Primary sternal osteomyelitis is a rare disease and is usually associated with any procedure of central venous catheterization. The case in question shows a young patient without comorbidities, no drug addiction nor prior vascular intervention, and the diagnosis was long delayed by the lack of reasonable suspicion of the pathology. We believe that if there had not been fast intervention, then this patient would have had a catastrophic or even fatal outcome. Treatment was mainly by immunosuppression with corticosteroids in sternal infection. This infection usually is polymicrobial, and *S. aureus*, which was cultured from our patient, is the most frequent one in these cases [5-7].

Primary sternal osteomyelitis treatment, as well as secondary, is based in surgical debridement and bacterial control with antibiotics. Most of the time, after these procedures, the wound is led to second intention healing or is followed by muscle flap rotation. The exact time to heal by second intention cannot be predicted because it depends on several factors, such as its evolution degree, and this technique can even progress to chronic wounds. The muscle flap rotation technique involves another surgical approach and the wound usually takes longer than 10 days to heal completely [8].

Although antibiotic therapy is the main part of osteomyelitis treatment, HBOT is being increasingly used for these conditions, especially refractory and chronic osteomyelitis [9,10].

However, there are still no studies in the literature demonstrating the benefits of the use of HBOT for the treatment of primary sternal osteomyelitis, considering it is a rare event.

## Conclusions

This case shows the difficulties of early diagnosis in primary sternal osteomyelitis and the risks of some wrong therapeutics.

The satisfying outcome of this patient reflects the quick action to promote surgical debridement and the use of antibiotics, which are both recommended treatments. The closure of the wound in 10 days after debridement suggests that the HBOT might have indirectly, but not conclusively, aided in the premature closure of the wound, avoiding a longer healing by second intention closure or muscle flap rotation.

Although there is no hard evidence that HBOT improves the final outcome of primary sternal osteomyelitis, this case shows a highly positive closure for the patient. In addition to well-established standard treatments such as surgical debridement and bacterial control, HBOT could be recommended as an adjunct tool of therapy for patients with primary sternal osteomyelitis [5,8,11].

## Consent

Written informed consent was obtained from the patient for publication of this case report and accompanying images. A copy of the written consent is available for review by the Editor-in-Chief of this journal.

## Competing interests

The authors declare that they have no competing interests.

## Authors’ contributions

TRN, JJRR and OF performed surgery and clinical evaluation of the patient; RFD and MNN analyzed and reviewed all examinations and the medical history of the patient regarding this pathology. All authors read and approved the final manuscript.
